# Performance Asymmetry, the Risk for Ankle Sprain, and the Influence of an Intervention Program in New Male Infantry Recruits

**DOI:** 10.3390/jcm14196887

**Published:** 2025-09-29

**Authors:** Michal Shenhar, Gali Dar, Aharon S. Finestone, Jeremy Witchalls, Gordon Waddington, Avi Shina, Nili Steinberg

**Affiliations:** 1 Department of Movement and Sport Sciences, Levinski-Wingate Academic College, Wingate Campus, Netanya 4290200, Israel; 2 Department of Physical Therapy, Faculty of Social Welfare and Health Studies, University of Haifa, Haifa 3103301, Israel; 3 Gray Faculty of Medical & Health Sciences, Tel Aviv University, Tel Aviv 6997801, Israel; 4 Department of Orthopedic Surgery, Shamir Medical Center, Be'er Ya'akov 7033001, Israel; 5 Research Institute for Sport and Exercise, University of Canberra, Bruce 2617, Australia; 6 Faculty of Health, University of Canberra, Bruce 2617, Australia; 7 Hebrew University Medical School, Jerusalem 9112102, Israel; 8 Israel Defense Forces Medical Corps, Ramat Gan 5251108, Israel

**Keywords:** interlimb asymmetry, ankle sprain, newly inducted infantry soldiers, intervention program

## Abstract

**Background**: Functional performance interlimb asymmetry may increase the risk of ankle sprains during basic military training. We aimed to (1) evaluate interlimb balance, agility, and ankle instability asymmetry in soldiers in infantry training as a risk factor for acute ankle sprains; (2) evaluate the effect of ankle sprains and sprain prevention exercise program on performance asymmetry. **Methods**: Newly inducted infantry soldiers were recruited from two induction cycles (intervention [INT] *n* = 365, control [CON] *n* = 421). Participants were assessed at the beginning of infantry basic training (T0) and after four months (T1) for anthropometrics, balance, agility, and perceived ankle instability, and were monitored for ankle sprains (SPRAIN/NO-SPRAIN). The INT group performed an ankle sprain prevention program 5 days/week × 5 min/day. **Results**: at T0 there were differences in interlimb asymmetry in Cumberland Ankle Instability Tool (CAIT) in SPRAIN soldiers in both groups (*p*-value < 0.001), and differences between the groups in Hexagon, Y-Balance Test (YBT) and CAIT (*p*-values 0.007, 0.002, 0.002, respectively). There was a decrease in interlimb asymmetry in Hexagon and YBT for SPRAIN soldiers in the INT group, and an increase in CAIT asymmetry in SPRAIN soldiers in both groups. Stepwise logistic regression did not find predictors for ankle sprains during training. **Conclusions**: The intervention program reduced interlimb asymmetry in balance and agility for soldiers who sprained their ankle during training. In these soldiers, CAIT asymmetry increased during training regardless of the intervention. Ankle sprain intervention programs should be implemented to reduce interlimb asymmetries in functional abilities and reduce the risk of injury.

## 1. Introduction

Ankle sprains are frequent injuries in active populations such as athletes and military personnel. They account for roughly 10% of all musculoskeletal injuries in the military and are a common cause of medical attrition [1,2]. The incidence rate of ankle sprains among military personnel is 27.9–58.4 per 1000 person-years [3,4]. Approximately 40% of those who experience at least one ankle sprain develop Chronic Ankle Instability (CAI), a condition characterized by recurrent sprains, episodes of pain and swelling, and the sensation of the ankle “giving way” [5]. Several systematic reviews and meta-analyses identified risk factors for ankle sprain injuries, including previous sprains, higher Body Mass Index (BMI), greater height, lower hip abduction strength, and lower balance ability [6,7]. Specifically in the military, previous sprains, higher BMI, lower proprioception ability, lower Achilles tendon quality, and lack of physical preparation before induction were found to be risk factors for ankle sprains [8].

Interlimb asymmetry in functional performance may potentially increase the risk of injury for both legs: the strong leg may sustain higher forces, whereas for the weak leg it might be difficult to sustain even average forces [9]. In athletes, it has been suggested that interlimb asymmetry may limit movement strategy, and as a result, the athlete may adopt a movement pattern that increases the risk of injury by accumulating fatigue or microtrauma [10]. Military personnel face demanding physical challenges that necessitate high level of functional performance capabilities such as dynamic balance and agility. These fundamental movement qualities are critical for operational effectiveness, enabling soldiers to navigate complex terrain, respond rapidly to changing tactical situations, and maintain stability under various environmental conditions [11]. However, when significant interlimb asymmetries exist in these functional capacities, soldiers may face elevated risk of acquiring ankle sprains. Various studies recognized interlimb asymmetry as a risk factor for musculoskeletal injuries. In female (but not male) basketball and floorball players (age > 21), asymmetry of hip abduction strength between the legs was found to be a risk factor for acute ankle injuries [12]. In male volleyball players asymmetry of quadriceps strength was found in a one-year injury follow-up study to be predictor for ankle sprains [13]. In young ballet dancers, interlimb asymmetry in several kinetic variables during double and single countermovement jumps was associated with as much as 69% higher risk for injuries of the lower back, pelvic area, and lower limbs [14]. In soldiers, Yavnai et al. (2021) reported that dynamic balance asymmetry along with impaired proprioception and perceived instability were risk factors for lower extremity musculoskeletal injuries [15].

Other studies investigated the influence of lower limb musculoskeletal injuries on interlimb asymmetry. For example, Yalfani & Raeisi (2022) found that elite basketball and soccer players with unilateral CAI needed a longer time for stabilization on the CAI side compared to the healthy side in double-leg landing task [16]. In young dancers, dynamic balance asymmetry was found to be associated with patellofemoral pain in a cross-sectional study performed on 132 dancers [17]. Tajdini et al. (2022) evaluated kinetic and electromyographic variables during walking in CAI and non-CAI individuals, and found that CAI subjects had greater asymmetry in vertical ground reaction force, as well as peroneus longus and gluteus medius activity [18]. In an English Premier League football club, over one third of the players reported previous ankle injuries, which were associated with interlimb asymmetries in the weight bearing lunge test (measuring ankle dorsiflexion, which is associated with ankle sprains) [19].

However, the relationship between balance, agility, and ankle instability asymmetries and ankle sprain risk remains unexplored in military infantry training. This study aimed to evaluate whether interlimb asymmetries are able to predict acute ankle sprains in soldiers and assess how sprains and a prevention program may affect performance asymmetry. We hypothesized that asymmetry would predict ankle sprains and that prevention interventions would reduce performance asymmetry.

## 2. Materials and Methods

Male participants between the ages of 18 and 21 years, with a mean ± standard deviation (SD) height 175 ± 6.6 cm, mean ± SD weight 74.9 ± 11.6 kg, and mean ± SD BMI 24.39 ± 3.4 were recruited from two induction cycles: An earlier cycle serving as the control (CON) group, and a later cycle, which was the intervention (INT) group. All participants were medically cleared for combat service. At the T0 assessment, 786 soldiers participated (421 in the INT group, 365 in the CON group). At the T1 assessment, 431 soldiers participated (183 in the INT group and 248 in the CON group). The reasons for attrition included medical or unsuitability to combat service, guard or kitchen duty at the day of the T1 assessment, and unexpected military schedule at the day of the T1 assessment.

Prior to consenting to participate in the study, all soldiers participated in an introductory session that took place at the training army base, in which the following information was provided: (1) participation was on a voluntary basis. (2) soldiers who chose not to participate or to withdraw from the study would not be penalized. (3) the soldiers’ commanders would not be exposed to the results of the study, and (4) soldiers could withdraw at any point from the study without having to provide reasons for withdrawal to the research team or the commanders. All participating soldiers signed an informed consent form.

### 2.1. Assessments

In each training cycle, two assessment sessions took place: the first assessment—at the beginning of training (T0), and the second assessment—at the end of training, approximately four months later (T1). At both sessions the following assessments were performed:

*Anthropometrics*—Height (Leicester Height Measure, Tanita) and weight (Electronic Personal Scale, Gala Home) were measured, and BMI was calculated (weight kg/height m^2^). Leg length was measured from the Anterior Superior Iliac Spine to the medial malleolus of both legs. The dominant leg was determined as the stance leg while kicking a ball [8].

**Figure 1:**
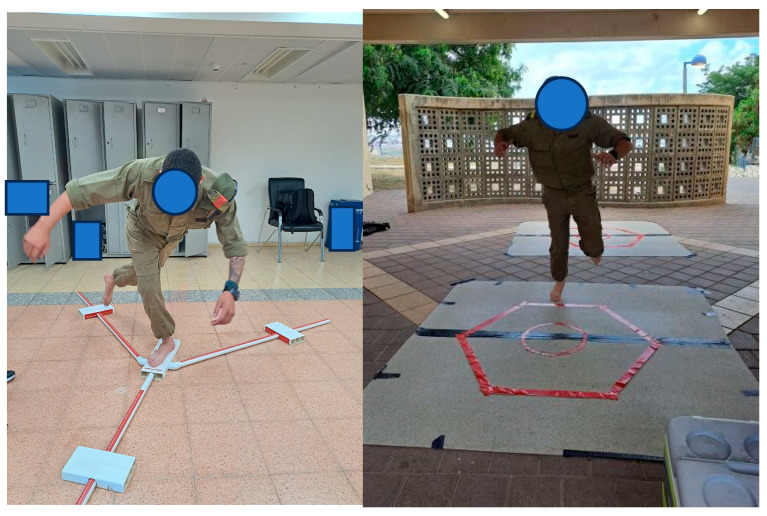
Y-balance test (**left**), Hexagon agility hop test (**right**).

*Hexagon agility hop test*—Used to evaluate agility. A 60 cm side hexagon was marked on the ground with a 40 cm diameter circle in its center. The participants were asked to stand on a single leg in the center of the circle, then hop forward beyond the side of the hexagon and back to the circle. The next hop was beyond the next side of the hexagon in a clockwise direction and back to the circle without turning. The participants continued hopping in a clockwise direction for 10 s. After one minute break to rest the leg, they performed the same hopping sequence on the same leg in a counterclockwise direction for 10 s. Then the participants performed the whole procedure on the other leg. In total, there were 20 s of hopping in different directions for each leg. The test score was the number of hops that the participant performed beyond the line of the hexagon in both directions combined for each leg (Figure 1) [20].

*Y-Balance test (YBT)*—Used to test dynamic balance. The YBT kit includes a central plate, and three rods marked with cm coming out of it in three directions: anterior, posteromedial (PM) and posterolateral (PL). On each rod there is a moveable box. The participants stood on the tested leg on the central plate and were instructed to move the plate on each rod with the other leg without leaning on it or kicking it. The distance to which they pushed the box in each direction was marked for each leg. Each leg’s composite score was calculated as (Anterior + PM + PL)/3 × (leg length) × 100 (Figure 1) [20].

*Cumberland Ankle Instability Tool (CAIT)*—This is a 9-item perceived ankle instability questionnaire. Each item has several statements, of which the participants had to mark the most suitable for each leg separately. A score of ≤25 was considered perceived ankle instability [20].

*Sprain during training*—Ankle sprains were clinically diagnosed during the training. Approximately once every 10–14 days, an experienced physiotherapist (MS) arrived at the army base to monitor the soldiers for ankle sprains. Soldiers who reported ankle injuries were clinically examined. The criteria for the diagnosis of an ankle sprain were: (1) fall, bad landing or any other incident that involved twisting of the ankle; (2) pain while running, walking or climbing up and down stairs; (3) hematoma, swelling or sensitivity in at least one of the following areas: anterior talofibular ligament, calcaneofibular ligament, posterior talofibular ligament, lateral malleolus, distal fibula [21]. Only the first sprain per participant and per leg was recorded, to avoid misclassifying residual pain from previous injuries as new sprain events.

*Intervention program*—The soldiers in the INT group performed ankle sprain prevention exercises (5 min/day × 5 days/week). The program included dynamic balance, strengthening, and agility exercises [22,23] that were performed while wearing army uniforms and boots. The exercises did not require equipment and thus they were suitable for performing in the army base as well as on field. The CON group continued their training routine. The intervention program comprised three sub-programs, each lasting 5 min. Every week the soldiers performed a sub-program in a consecutive order, so that all the soldiers performed the same program each week. The exercises comprising the programs were: (1) single leg squat; (2) the “clock” balance exercise; (3) square hops (clockwise/counter clockwise directions); (4) single leg step-up (forward or sideways); (5) single leg pendulum balance (forward-backward of sideways); (6) hop and stop (clockwise and counter clockwise directions); (7) bilateral heel raise; (8) forward/backward/sideways hops; and, (9) closed-eye single leg squat balance.

### 2.2. Data Analysis

In each group (INT, CON), the soldiers were divided into SPRAIN (those who sprained their ankle during training) and NO-SPRAIN (those without ankle sprains during training). Absolute value of interlimb differences (non-dominant leg minus dominant leg) were calculated for balance (YBT anterior, PL and PM directions, and composite score), agility (Hexagon hop test), and ankle instability (CAIT) at T0 and T1. A 2-way Analysis of Variance (ANOVA) was performed to assess the differences in asymmetry between the SPRAIN and NO-SPRAIN soldiers in the INT and CON groups at T0. Repeated measures (T0/T1 assessments) ANOVA with two between-subjects variables [group (INT, CON) and sprain during training (yes/no)] was conducted to evaluate whether there was an effect of the intervention on the differences in interlimb asymmetry from T0 to T1 assessments. Stepwise logistic regression was performed with the T0 interlimb differences measures in YBT (YBT anterior, PL and PM directions, and composite score), Hexagon, and CAIT to evaluate whether asymmetry in those variables can predict ankle sprains during training.

*Sample size calculation:* This interventional study is part of a bigger project aimed at reducing the incidence of ankle sprains in infantry soldiers from 15% to 8%. The sample size was calculated based on previously reported ankle sprain rates among soldiers, which range from 15% to 18% [24,25]. To ensure a statistical power of 0.80 and a significance level of α = 0.05, a minimum of 650 participants in total were required.

Statistical analysis was performed using JASP version 0.95 [26]. A difference marked by a *p* ≤ 0.05 was considered statistically significant in all statistical tests.

## 3. Results

**Figure 2:**
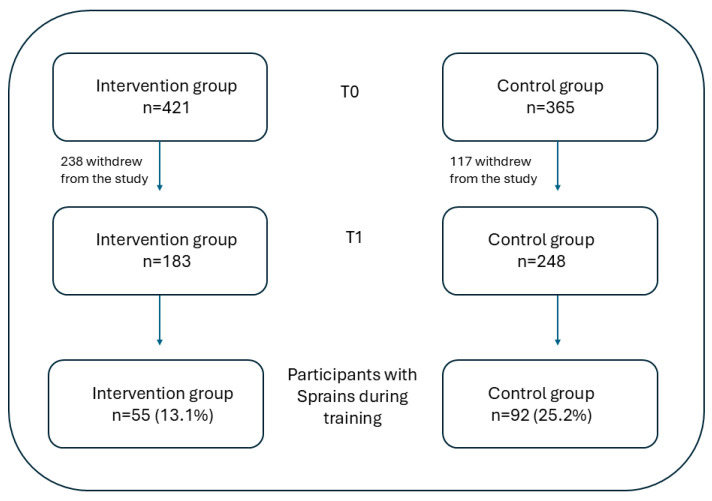
Flow chart of the number of participants in the two study groups at T0 (first assessment) and T1 (second assessment), and the number and percentage of participants with sprains acquired during training. Note: the percentage is calculated out of the total number pf participants in the respective group at T0.

Of the 786 soldiers who participated in the T0 assessments, 147 were diagnosed with an ankle sprain during training (55 [13.1%] in INT, 92 [25.2%] in CON, X^2^ *p* < 0.001) (Figure 2).

The mean ± SD values for the anthropometric and asymmetry measures at T0 and T1 are provided in Table 1. At T0, the mean weight of the SPRAIN soldiers in both the INT and CON groups was higher than that of the NO-SPRAIN soldiers, and their mean BMI greater (*p* = 0.048 and 0.015, respectively). In the asymmetry measures, the SPRAIN soldiers had a greater asymmetry in CAIT (sprain effect *p* < 0.001). There were differences between the INT and CON groups in asymmetries of Hexagon (greater in the INT group, *p* = 0.007), YBT posteromedial direction (greater in the CON group, *p* = 0.028), YBT composite score (greater in the INT group, *p* = 0.002), and CAIT (greater in the INT group, *p* = 0.002). Group × sprain interactions were found for CAIT (F_(1726)_ = 8.551, *p* = 0.004) and Hexagon (F_(1717)_ = 4.883, *p* = 0.027), where the asymmetry was greater in the INT group.

Table 2 presents the group effect, sprain effect and interaction findings of the repeated measures ANOVA. In Hexagon, the asymmetry was greater in the INT versus the CON group (group effect *p* = 0.028). Sprain effect (*p* = 0.045) and time × group × sprain interaction (F_(1373)_ =4.620, *p* = 0.032) were also found, where the asymmetry decreased for the SPRAIN soldiers in the INT group from T0 to T1.

In YBT PL direction there was a reduction in asymmetry from T0 to T1 in the SPRAIN soldiers in the INT group (time effect *p* = 0.049). A time × group × sprain interaction (F_(1394)_ =5.913, *p* = 0.015) was also found for this variable where the asymmetry decreased for the SPRAIN soldiers in the INT group from T0 to T1.

In CAIT, we found time effect (*p* < 0.001) and a sprain effect (*p* < 0.001), where in both groups the asymmetry increased for the SPRAIN soldiers but not for the NO-SPRAIN soldiers.

The results of the stepwise logistic regression analysis are presented in Table 3. Interlimb asymmetry in agility (Hexagon) and perceived ankle instability (CAIT) entered the regression for ankle sprain prediction. However, the model was able to identify only 0.8% of the sprains (Nagelkerke R^2^ = 0.038).

## 4. Discussion

The main finding of this study was that the intervention program applied in the INT group reduced asymmetry in Hexagon and YBT PL direction for the SPRAIN soldiers but not for the NO-SPRAIN soldiers. In the CON group there were no significant changes in YBT and Hexagon asymmetry. Sprains acquired during training increased perceived ankle instability asymmetry (CAIT) for the SPRAIN soldiers in both the INT and CON groups. Balance, agility, and ankle instability interlimb asymmetry were not identified as risk factors for ankle sprains during basic infantry training according to the stepwise logistic regression analysis.

There are inconsistencies in the literature regarding the association between YBT asymmetries and the risk of lower extremity injuries [9]. Our findings, showing no association between YBT asymmetry and risk of injury, are similar to those of Lisman et al. (2021), and Brummit et al. (2018), who also found no association between the two parameters in high school athletes and in male collegiate basketball players, respectively [27,28]. In contrast, Smith et al. (2015) identified asymmetry in the anterior direction of the YBT as a risk factor for lower extremity injury [29]. In an earlier study, Plisky et al. (2006) reported that the asymmetry of >4 cm in the anterior reach direction on the Star Excursion Balance Test (a similar test to the YBT) was a risk factor for lower extremity injury among male and female high school basketball players [30]. When concerning ankle sprains, anterior reach asymmetry measure in preseason was greater in baseball players who sprained their ankle during the season [31]. while asymmetries in all YBT directions were found to be risk factors for ankle sprains in firefighters [32]. Lower dynamic balance ability can be related to lower stiffness of the ankle joint, indicating weaker around-the-ankle muscle strength [33], which could be influenced by an ankle sprain injury [5]. Following injury, weakening of this ligament allows excessive forward displacement of the talus, which subsequently restricts dorsiflexion range of motion. This mechanical limitation, documented in post-ankle sprain populations, may ultimately compromise balance function [34].

Perceived ankle instability is a common complaint after an ankle sprain, especially among those who develop CAI [5]. The increase in the perception of ankle instability after an ankle sprain injury may be due to ligamentous damage, and sensory-motor changes that occur as a result of inflammatory and pain mediation processes following the sprain, that in turn can cause sensory-perceptual and motor-behavioral impairments [5]. The CAIT questionnaire that was used in this study to evaluate perceived ankle instability, was developed in order to report instability of one ankle independently of the other [35]. In spite of this, the majority of publications quantifying ankle instability, treat CAIT results as one, not distinguishing between the scores of the two legs [36,37,38]. There are, however, several studies that provide separate assessments for the injured and uninjured legs. For example, in one study, individuals with CAI reported that the involved leg felt less stable than the uninvolved leg in a series of hop tasks [35]. This is similar to the results of our study, showing that while the intervention reduced asymmetry in the YBT and Hexagon tests for the SPRAIN soldiers in the INT group, it did not affect CAIT asymmetry. The difference in CAIT scores increased in the SPRAIN soldiers in both the INT and CON groups, which may be due the score of the injured leg having decreased (the perception of ankle instability increased because of the sprain). Lower CAIT scores (but not interlimb asymmetry scores) were found to be predictors for lower extremity injuries in football players [39], college students [40], and soldiers [41].

Regarding agility, which we measured using the Hexagon hop test, research on asymmetry mostly focuses on return to sport assessments rather than as injury-predicting factor [42]. The asymmetry in Hexagon in the current study decreased following an intervention program in the SPRAIN soldiers but not in the NO-SPRAIN soldiers of the INT group. These results are in agreement with a previous study performed on young soccer players, that showed that an 8-week intervention consisting of explosive power training reduced interlimb agility asymmetry [43]. Our results showed that asymmetry in agility cannot be considered a risk factor for ankle injury. However, in a study in volleyball players, interlimb asymmetry in the T-test for agility was found to be a risk factor for non-contact lower limb injuries [44]. Balance, agility, and ankle instability are associated with reduced proprioception that was found in individuals with CAI [45]. Effective proprioception is critical for maintaining stability during activities such as traversing irregular surfaces—a common requirement in military operations. Ankle sprain injuries can compromise proprioceptors located in the ankle’s periarticular ligaments and muscle spindles, leading to impaired proprioceptive function [45].

The intervention program used in the current study consisted of equipment-free balance, agility, and strength exercises, enabling soldiers to perform the training both on base and in field environments. A well-documented ankle sprains prevention program is the FIFA 11+ program, that was designed for reducing injuries in football players [46]. The FIFA 11+ program, which includes running, balance and strength exercises, is performed as part of the warmup and has been proven to reduce ankle injuries [47]. The exercises performed in our study are in agreement with the FIFA 11+ intervention program, as well as other programs described in systematic reviews on ankle sprain prevention exercise programs [23,48].

In the current study, interlimb asymmetries in ankle agility and balance, and ankle instability perception were not found as predictive factors for ankle sprains acquired during training. A possible explanation for this could be that other factors such as fatigue due to military training [49], unexpected movements as part of the training, and/or training on uneven terrain carrying loads [50], were more dominant in their association with ankle sprains compared to baseline interlimb asymmetries. However, these were not assessed in this study, and we propose that future studies investigate them as potential predictive factors for ankle sprains during training.

### 4.1. Limitations

There are several limitations to this study. Firstly, we did not collect medical history that may have influenced the soldiers’ physical abilities. Notably, however, the population of infantry new recruits is medically cleared by the army medical authorities before induction, minimizing the likeliness of medical history with potential to affect the results of the study. Data about previous ankle sprains and about the soldiers’ fitness regime prior to participation in this study was not collected either. In addition, we did not monitor the participants’ compliance with the intervention program. Generalization of these results should be made cautiously, as the population in this study was homogenous in terms of gender and age group and was considered healthy.

### 4.2. Clinical Implications

Military commanders and medical staff should be aware of the balance, agility, and perceived ankle instability asymmetries and their potential relation to ankle injuries. Based on our results, providing intervention programs to soldiers who suffer ankle sprains in their infantry training may reduce asymmetry in balance and agility, possibly leading to a reduced risk for reinjury.

## 5. Conclusions

This study showed that an ankle sprain preventive exercise program in soldiers was able to decrease balance and agility interlimb asymmetry, which are potential risk factors for lower limb injury. However, in the perception of ankle instability, the interlimb asymmetry in the soldiers with ankle sprains increased regardless of whether they performed the preventive program or not. Interlimb asymmetry in balance, agility, and perceived ankle instability was not a predictor for ankle sprains during infantry training. As ankle sprains are multifactorial, future studies should investigate internal asymmetry as part of a complex of variables that could be associated with this injury in military training. An ankle sprain preventive exercise program is recommended to decrease interlimb asymmetry during infantry basic training. Future studies should investigate interlimb asymmetries and their association with ankle sprains and broaden the population segments being tested.

## Figures and Tables

**Table 1:** Mean ± SD values for anthropometric and asymmetry measures of participants at T0 presented by the SPRAIN/NO-SPRAIN during training and the INT/CON groups.

Anthropometric/Asymmetry Measure	Intervention		Control	
	SPRAIN n = 55	NO-SPRAIN n = 366	SPRAIN n = 92	NO-SPRAIN n = 273
Height	174.73 ± 6.4	175.04 ± 6.6	175.43 ± 7.1	175.55 ± 6.4
Weight ^&^	76.34 ± 12.4	74.35 ± 10.5	77.12 ± 12.6	74.60 ± 12.4
BMI ^&^	25.05 ± 3.6	24.28 ± 3.1	25.04 ± 3.7	24.17 ± 3.5
Hexagon asymmetry ^$@^	2.20 ± 1.9	2.09 ± 1.7	1.28 ± 1.2	1.99 ± 1.8
YBT Ant asymmetry	5.83 ± 4.2	5.26 ± 5.0	4.60 ± 4.1	5.71 ± 6.0
YBT PL asymmetry	8.28 ± 8.4	6.76 ± 6.2	7.09 ± 6.2	7.39 ± 6.7
YBT PM asymmetry ^$^	6.68 ± 6.1	7.77 ± 7.3	8.48 ± 6.7	9.11 ± 7.3
YBT Comp asymmetry ^$@^	9.67 ± 19.4	5.68 ± 7.9	4.72 ± 3.8	5.64 ± 6.1
CAIT asymmetry ^$&@^	2.86 ± 4.5	0.90 ± 2.1	1.26 ± 3.6	0.83 ± 2.4

^$^ refers to group effect; ^&^ refers to sprain effect; ^@^ refers to interactions. Ant = anterior, BMI = Body Mass Index, CAIT = Cumberland Ankle Instability Tool, Comp = Composite score, PL = posterolateral, PM = posteromedial, YBT = Y-Balance test.

**Table 2:** The findings of 2-way ANOVA with two between-subjects variables (group [CON/INT] and sprain during training (yes/no) in study participants.

Asymmetry Measure	Intervention				Control			
	SPRAIN n = 55		NO-SPRAIN n = 366		SPRAIN n = 92		NO-SPRAIN n = 273	
	T0	T1	T0	T1	T0	T1	T0	T1
Hexagon ^$&@^	2.30 ± 1.7	1.44 ± 1.1	2.10 ± 1.9	2.13 ± 2.0	1.29 ± 1.2	1.52 ± 1.8	1.97 ± 1.6	1.71 ± 1.4
YBT Ant	5.44 ± 3.5	4.65 ± 4.3	4.96 ± 4.8	4.67 ± 3.8	4.47 ± 3.7	5.30 ± 4.4	5.39 ± 4.9	5.57 ± 9.1
YBT PL ^#@^	10.30 ± 10.5	6.22 ± 5.5	6.65 ± 6.3	6.39 ± 5.6	7.20 ± 6.4	7.98 ± 6.5	7.53 ± 7.1	6.56 ± 5.3
YBT PM	6.52 ± 5.2	6.17 ± 4.7	7.63 ± 7.4	6.94 ± 6.5	8.65 ± 6.5	6.64 ± 5.4	8.50 ± 6.7	7.09 ± 5.9
YBT Comp	8.21 ± 14.3	6.84 ± 8.6	5.10 ± 5.0	6.19 ± 13.5	4.65 ± 3.5	5.28 ± 4.7	5.40 ± 6.7	5.08 ± 4.7
CAIT ^#&^	1.37 ± 1.5	5.16 ± 5.7	0.89 ± 1.8	1.49 ± 3.9	1.08 ± 2.4	3.26 ± 5.8	1.09 ± 2.7	1.08 ± 2.7

^#^ refers to time effect; ^$^ refers to group effect; ^&^ refers to sprain effect; ^@^ refers to interactions; T0 refers to the first assessment; T1 refers to the second assessment. Ant = anterior, CAIT = Cumberland Ankle Instability Tool, Comp = Composite score, PL = posterolateral, PM = posteromedial, YBT = Y-Balance test.

**Table 3:** Results of a stepwise logistic regression analysis for asymmetry variables associated with ankle sprains during basic infantry training in study participants.

Variable	Estimate	Standard Error	Odds Ratio	Wald Statistic	*p*-Value
Hexagon Diff	−0.177	0.066	0.838	7.198	0.007 *
CAIT Diff	0.093	0.033	1.097	8.159	0.004 *

* Statistical significance was considered at *p* ≤ 0.05. CAIT = Cumberland ankle instability tool, Diff = difference.

## Data Availability

There are no additional data available due to participants’ confidentiality.

## Abbreviations

The following abbreviations are used in this manuscript:

CAIT	Cumberland Ankle Instability Tool
YBT	Y-balance test
INT	intervention group
CON	control group
CAI	Chronic Ankle Instability
